# Efficacy and safety of tacrolimus treatment for neuromyelitis optica spectrum disorder

**DOI:** 10.1038/s41598-017-00860-y

**Published:** 2017-04-11

**Authors:** Bo Chen, Qian Wu, Gaotan Ke, Bitao Bu

**Affiliations:** 1 Department of Neurology, Tongji Hospital of Tongji Medical College, Huazhong University of Science and Technology, Wuhan, Hubei China; 2 Department of Radiology, Tongji Hospital of Tongji Medical College, Huazhong University of Science and Technology, Wuhan, Hubei China

## Abstract

Neuromyelitis optica spectrum disorder (NMOSD) is a severe inflammatory autoimmune disease that mainly involves the optic nerves and spinal cord, causing blindness and paralysis. Although some immunosuppressants such as rituximab and azathioprine have proven to be effective in relapse prevention, the high costs or intolerable adverse events preclude their wide application. Thus, we have conducted a retrospective study in 25 NMOSD patients who were treated with tacrolimus, an immunosuppressant with high efficacy and good tolerance in other autoimmune diseases, to assess its efficacy and safety in NMOSD treatment during the last five years (2011–2016). The results revealed that tacrolimus could reduce the relapse rate by 86.2% and improve the Expanded Disability Status Scale (EDSS) scores (4.5 vs 2.3; P < 0.001) significantly. Relapses in tacrolimus treatment were associated with serum titers of aquaporin 4 antibody (AQP4-IgG) (P = 0.028). Further Cox proportional analysis demonstrated that patients with high titers of AQP4-IgG (≥1:64) had a significantly higher risk of relapse than those with low titers after tacrolimus therapy (HR:5.665; CI_95_: 1.012–31.705; P = 0.048). Tacrolimus tended to be superior to azathioprine (29 patients) in terms of efficacy and safety during the same period. Our study suggests that tacrolimus may be another promising immunosuppressant for NMOSD.

## Introduction

Neuromyelitis optica spectrum disorder (NMOSD) is an inflammatory autoimmune disease of the central nervous system (CNS), predominantly affecting the optic nerves and spinal cord. Recurrent attacks of the vulnerable CNS structures in patients with NMOSD lead to more severe and devastating deficits in the visual and motor functions than those in patients with multiple sclerosis (MS). Pathological studies in NMO patients have demonstrated inflammation with macrophage predominance, immunoglobulin deposition, perivascular granulocytes and eosinophils, as well as extensive axonal loss in the spinal cord and optic nerve damage^1^. Specific IgG antibodies that are directed against water channel aquaporin 4 (AQP4-IgG) exist in approximately 70% of patient cases, which is distinct from MS patients. Patients with NMOSD require immunosuppressive therapies to reduce or prevent relapses, but some immunomodulatory therapies such as interferon-beta used for MS appear to exacerbate NMO^2, 3^. Unfortunately, there have been no published randomized controlled treatment trials in NMO/NMOSD, though a variety of immunosuppressants including azathioprine, mitoxantrone, mycophenolate, rituximab, and methotrexate, were reported to be effective in relapse prevention in small-sized studies^4–14^. However, more than 50% of patients who received azathioprine or methotrexate had at least 1 relapse while undergoing therapy^11, 14^. Although rituximab is considered to be a promising agent for NMOSD^11^, with 67% of patients being relapse-free, the considerable cost and monitoring of CD19 and CD20 cell counts by flow cytometry limit its availability. Therefore, attacks of the disease still occurred despite use of these immunosuppressive agents.

Tacrolimus, another immunosuppressant that blocks T cell activation by specifically inhibiting calcineurin, is widely used in organ transplantation and autoimmune diseases such as myasthenia gravis, inflammatory myopathy, ulcerative colitis and lupus nephritis^15–18^. Nevertheless, tacrolimus is rarely used in the treatment of NMOSD. Therefore, in this study, we aimed to evaluate the efficacy and safety profile of tacrolimus treatment in Chinese patients with NMOSD.

## Results

### Baseline demographic and clinical data

Twenty-five patients with NMOSD who received tacrolimus were included. Table 1 summarizes the demographic and baseline clinical characteristics of these patients. Twenty-four patients (96%) were treated with 2–3 mg/d of oral tacrolimus, and one young child was treated with 1 mg/d. Simultaneously, 15 patients (60%) were treated with concomitant prednisone at a maintenance dose range of 2.5 to 20 mg/d for more than 6 months. Intravenous immunoglobulin G (IVIG) and plasmapheresis were used as rescue therapies during acute attacks in 4 patients with severe neurological deficits. One was previously diagnosed with MS and received interferon-beta for one year before tacrolimus treatment. Another 5 patients who took other immunosuppressants such as cyclophosphamide (n = 1), azathioprine (n = 3), and mycophenolate mofetil (n = 1), were switched to tacrolimus because of relapses or severe adverse effects of the agents.

**Table 1:** Demographic characteristics of the patients included.

Characteristics	Value
Patients, No.	25
Female sex, No. (%)	23 (92)
Age at onset, median (Range), y	31 (6–55)
Duration before receiving tacrolimus, median (Range), mo	16 (1–98)
Duration of tacrolimus, median (Range), mo	11 (6–34)
AQP4-IgG serostatus	
AQP4-IgG positivity, No. (%)	22 (88)
Titer of AQP4-IgG <1:64 or none, No. (%)	14 (56)
Titer of AQP4-IgG ≥1:64, No. (%)	11 (44)
Previous immunotherapy, No. (%)	
Intravenous immunoglobulin G	2 (8)
Interferon-beta	1 (4)
Plasmapheresis	2 (8)
Azathioprine	3 (12)
Cyclophosphamide	1 (4)
Mycophenolate mofetil	1 (4)

AQP4-IgG: aquaporin 4 immunoglobulin G.

### Treatment effect

**Figure 1:**
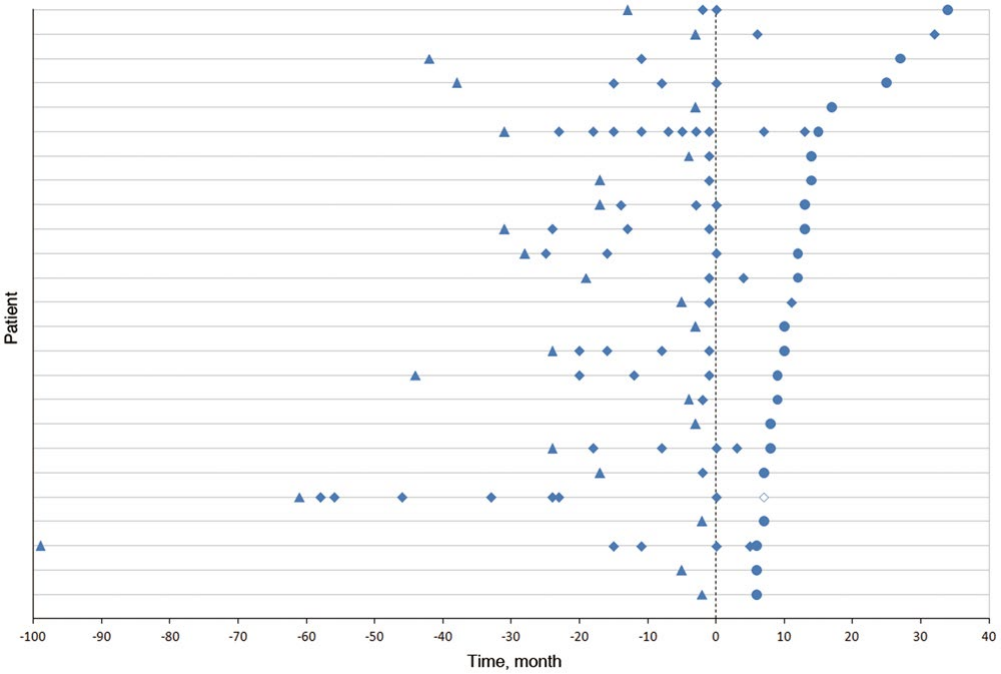
Relapses in Patients With Neuromyelitis Optica Spectrum Disorder (NMOSD) Before and After Tacrolimus Treatment. Figure dipicts relapses before and after treatment with tacrolimus in 25 NMOSD patients, showing a significant reduction in relapse rate (86.2%), with only 1 death. Each row of symbols on the y-axis represents a patient. All the patients were prescribed tacrolimus at time 0.  First attack.  Relapses.  Last follow-up.  Death.

Figure 1 illustrates relapses before and after the treatment with tacrolimus in 25 patients (details are given in Supplementary Table S1). Seven patients (28%) had at least 1 relapse while undergoing therapy, with a total of 9 relapses among them, whereas 18 (72%) were relapse-free (Table 2). Of all the 9 relapses, 6 (66.7%) occurred within 7 months after the initiation of tacrolimus therapy. The treatment with tacrolimus also contributed to a reduction of annualized relapse rate (ARR) in 23 of 25 patients (92%). The ARR before the tacrolimus treatment was 2.9, and it decreased to 0.4 after the therapy, amounting to a significant reduction of 86.2% (P < 0.001). The Expanded Disability Severity Scale (EDSS) scores were improved significantly, from a mean of 4.5 before treatment to 2.3 at the last follow-up (P = 0.001). Twenty-four patients (96%) experienced an improved or stabilized EDSS score.

**Table 2:** Treatment Efficacy in all the Patients who received tacrolimus.

	Before tacrolimus treatment	After tacrolimus treatment	Change from pretreatment to posttreatment, %	P-value^a^
Mean ARR	2.9	0.4	86.2	<0.001
Mean EDSS	4.5	2.3	48.9	0.001
Relapse free, %	72			
Improved ARR, %	92			
Improved or Stabilized EDSS Score, %	96			

ARR: annualized relapse rate; EDSS: Expanded Disability Severity Scale.

^a^Wilcoxon signed rank test.

Twenty patients (80%) took tacrolimus as their initial immunosuppressant (including those who received immunomodulatory therapy like interferon-beta, IVIG, and plasmapheresis). Among them, the ARR and EDSS scores decreased from 3.0 to 0.4 (P < 0.001), and 4.2 to 2.2 (P < 0.001), respectively (Table 3). The reduced ARR occurred in 19 patients (95%), 14 of whom were relapse-free. The EDSS scores were improved or stabilized in all the patients.

**Table 3:** Treatment Efficacy in Patients who received tacrolimus as initial immunosuppressant.

	Before tacrolimus treatment	After tacrolimus treatment	Change from pretreatment to posttreatment, %	P-value^a^
Mean ARR	3.0	0.4	86.7	<0.001
Mean EDSS	4.2	2.2	47.6	<0.001
Relapse free, %	70			
Improved ARR, %	95			
Improved or Stabilized EDSS Score, %	100			

ARR: annualized relapse rate; EDSS: Expanded Disability Severity Scale.

^a^Wilcoxon signed rank test.

**Figure 2:**
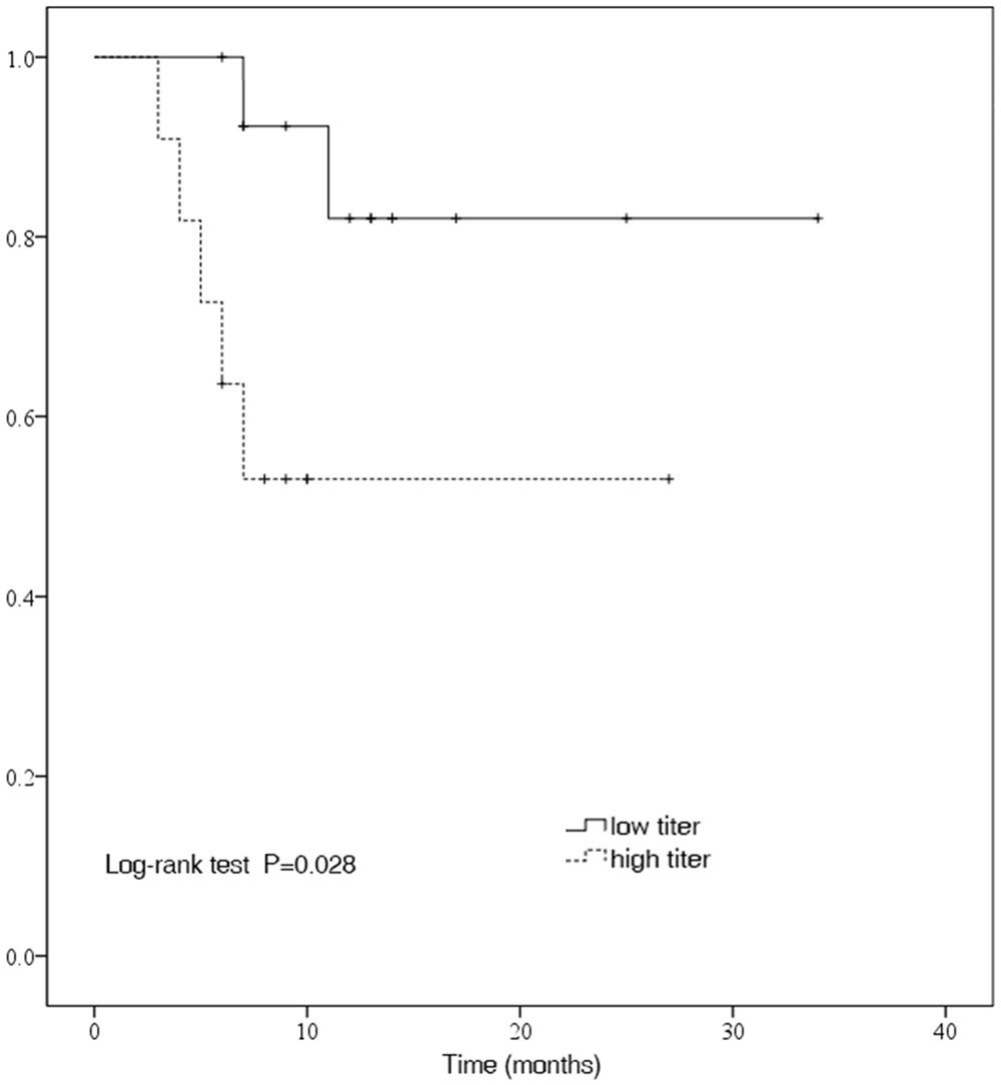
Kaplan–Meier estimates probability of being free of any relapse by AQP4-IgG serostatus after tacrolimus treatment. Kaplan–Meier analysis revealed that there was a significant difference between the high titer (≥1:64) group of aquaporin 4 antibody (AQP4-IgG) in the serum and the low titer group(<1:64 or none) regarding relapses while undergoing tacrolimus therapy (P = 0.028 by log-rank test).

The Kaplan–Meier curves for the group analysis according to the titers of AQP-4 antibody are shown in Fig. 2. The estimate of cumulative proportion of relapse-free patients was 0.82 in the low titer group and 0.53 in the high titer group after tacrolimus treatment. There was statistical significance between the two different groups based on the titers, regarding the occurrences of the first relapse (P = 0.028 by log-rank test).

In the univariate Cox proportional hazards regression model, relapses after treatment were associated with the titer of AQP-4 antibody before tacrolimus treatment (HR:5.665; P = 0.048). However, multivariate analysis revealed that the difference did not reach statistical significance (P = 0.061) (details are given in Table 4). The number of attacks prior to tacrolimus treatment seemed to be associated with relapse, but there was no statistical difference in either univariate (P = 0.060) or multivariate analysis (P = 0.071).

**Table 4:** Cox proportional hazards analysis of relapse.

Variables	HR (95% CI)	p-value	Adjusted HR (95% CI)	p-value
Age at onset	0.987 (0.936–1.041)	0.632	1.011 (0.946–1.081)	0.739
Pre-EDSS	0.994 (0.615–1.607)	0.980	1.109 (0.674–1.825)	0.683
AQP4-IgG titer	5.665 (1.012–31.705)	**0.048**	5.671 (0.922–34.876)	0.061
Gender	—	0.582	—	>0.99
Number of attacks before treatment	1.310 (0.989–1.736)	0.060	1.360 (0.974–1.899)	0.071

HR: hazard ratio; CI: confidence interval; Pre-EDSS: Expanded Disability Severity Scale before tacrolimus treatment.

In the univariate Cox proportional hazards regression model, the titer of AQP-4 antibodies before tacrolimus treatment was associated with relapse after treatment (HR: 5.665 95% CI: 1.012–31.705, P = 0.048). However, multivariate analysis revealed that the difference did not reach statistical significance (P = 0.061). The number of attacks prior to tacrolimus treatment, gender, EDSS before treatment, and age at onset were not associated with relapse when tacrolimus treatment was commenced in either univariate or multivariate analysis.

### Comparison between patients who received tacrolimus and those who received azathioprine

Twenty-nine NMOSD patients received azathioprine during the same period. Table 5 demonstrates a comparison between patients who received tacrolimus and those who received azathioprine. Azathioprine could reduce the ARR and EDSS score by 77.8% and 9.3%, respectively. However, azathioprine therapy provided a relatively lower relapse-free rate and a significantly lower rate of improved or stabilized EDSS score than tacrolimus treatment did (relapse-free rate, 48% vs 72%, P = 0.1; rate of improved or stabilized EDSS score, 62% vs 96%, P = 0.003).

**Table 5:** Comparison between patients who received tacrolimus and those who received azathioprine.

Characteristics	Tacrolimus	Azathioprine	P-value
Patients, No.	25	29	
Age at onset, median, y	31	38	0.142^a^
Treatment duration, median, month	11	16	0.220^a^
Pre-ARR vs Post-ARR	2.9 vs 0.4	2.7 vs 0.6	
Change from pretreatment to posttreatment, %	86.2	77.8	
Pre-EDSS or Post-EDSS	4.5 vs 2.3	4.3 vs 3.9	
Change from pretreatment to posttreatment, %	48.9	9.3	
Relapse free, %	72	48	0.100^b^
Improved or stabilized EDSS score, %	96	62	**0.003** ^b^
Total adverse events, No.	4	9	0.223^b^
Severe adverse events^c^, No.	1	7	0.056^b^

ARR: annualized relapse rate; EDSS: Expanded Disability Severity Scale.

^a^Mann-Whitney U test.

^b^Fisher’s exact test.

^c^severe adverse events included agranulocytosis, liver damage, and death etc.

Azathioprine could reduce the ARR and EDSS score by 77.8% and 9.3%, respectively. However, in comparison with tacrolimus treatment, azathioprine therapy provided a relatively lower relapse-free rate and a significantly lower rate of improved or stabilized EDSS score (relapse-free rate, 48% vs 72%, P = 0.1; rate of improved or stabilized EDSS score, 62% vs 96%, P = 0.003). Azathioprine group exhibited considerably higher adverse events occurrence of total adverse events (31%) and severe adverse events (24%) than observed in tacrolimus group (16% and 4%, respectively).

### Adverse events

Generally, treatment with tacrolimus was well tolerated in 24 patients, except one death. The dead patient was treated with 2000 mg/d of mycophenolate mofetil initially, and after having experienced 3 relapses over the next two and half years, she was switched to tacrolimus. Nevertheless, she died from an acute severe generalized infection in the seventh month of tacrolimus treatment. Of the remaining 24 patients, 3 still adhered to treatment in spite of mild adverse events, including hand tremor (n = 1, 4%), transient skin rash (n = 1, 4%), and herpes zoster (n = 1, 4%). In contrast, 9 patients (31%) who received azathioprine developed adverse events, including 7 severe adverse events such as agranulocytosis (n = 4, 14%), liver damage (n = 2, 7%), and death (n = 1, 3%) (Table 5).

## Discussion

This retrospective cohort study provided the fact that tacrolimus alone or in combination with oral prednisone was able to significantly reduce the relapse rate (2.9 vs 0.4; P < 0.001) and improve disability (4.5 vs 2.3; P = 0.001) for a considerable duration. Meanwhile, the drug was also well tolerated and relatively easily accessible. In our study, 18 of 25 patients (72%) were relapse-free after tacrolimus treatment, and 96% had improved or maintained disability, although this analysis was performed in a relatively small number of patients. The efficacy was comparable with that of mycophenolate mofetil in NMOSD patients^7, 11^. In a study by So-Young *et al*., mycophenolate provided a significant reduction in ARR (pre-ARR vs post-ARR, 2.6 vs 0.5; P < 0.001) and in EDSS score (pre-EDSS vs post-EDSS, 3.2 vs 2.7; P = 0.005) in 58 patients with a median treatment duration of 20.4 months, and 91% of patients had an improved or stabilized EDSS score^7^. Fourteen patients experienced mild adverse events such as skin rash, fatigue, and mild hair loss, with one discontinuation^7^. Another study by Mealy *et al*. reported that 75% of the 28 patients who received mycophenolate were relapse-free^11^. Although several studies have proved azathioprine to be effective for NMO^19, 20^, insufficient therapeutic efficacy and frequent adverse effects preclude its wide application. Our data showed a tendency that azathioprine was inferior to tacrolimus in the achievement of the state of relapse-free (48% vs 72%). Importantly, azathioprine exhibited a significantly lower rate of improvement of neurological disability relative to tacrolimus (62% vs 96%; P = 0.003). Also, azathioprine therapy contributed to a higher occurrence of severe adverse event (24%) than tacrolimus treatment (4%).

It needs to be explored that whether or not the previous immunotherapies such as IVIG, plasmapheresis, and other immunosuppressants exert some effects on the effectiveness of tacrolimus. In fact, plasmapheresis is commonly used as an adjunctive therapy during acute relapse of NMOSD patients, especially when they presented severe neurological deficits and were unresponsive to the pulse methylprednisolone^21^. Whether plasmapheresis or IVIG used in the early stage of tacrolimus treatment causes long-term effect on relapse or functional disability remains elusive, and no relevant study is available. The possibility that other immunosuppressants preceding tacrolimus may influence the observation could be trivial, because on one hand, the 5 patients who took the agents before tacrolimus had been experiencing relapses, and on the other hand, last evaluation of the patients was performed at least 6 months later, long enough for the agents to be eliminated from the bodies.

In our study, we found that 6 out of 9 relapses occurred during the first 7 months after the initiation of tacrolimus treatment, which means that tacrolimus may take several months to exert therapeutic effects in some cases. This observation was also noted by Tanaka *et al*.^22^, who found that the relapse in patients taking tacrolimus developed in the first few months. Accordingly, concurrent oral corticosteroid was recommended during early stages of tacrolimus treatment by Tanaka *et al*.^22^.

Interestingly, the titer of AQP4-IgG in the serum before tacrolimus treatment tended to be an influential factor in predicating relapse after the initiation of treatment, with a significant difference in the log-rank test (P = 0.028) and univariate Cox proportional hazards analysis (P = 0.048). However, tacrolimus was still able to reduce relapse rates in these patients. It might be inferred that patients with high titers of AQP4-IgG had high risks of relapse, even if they had been treated with immunosuppressants. In addition, we found that the number of attacks prior to treatment may not affect the relapse rate thereafter. Based on our study, we still strongly recommend that the patients who are diagnosed with NMOSD should be treated with immunosuppressants as soon as possible, in consensus with the study by Jiao Y *et al*., which suggested that early initiation of immunotherapy may lead to a more favorable motor outcome^23^. Our study did not disclose the association of EDSS scores before treatment and age at onset with relapse rates after the therapy (Table 4).

Another major concern is the safety of the drugs for NMOSD. Tacrolimus has been shown to be safe and well-tolerated in lupus nephritis^18^, myasthenia gravis^15, 24^, inflammatory myopathies^16^, and organ transplantation patients. Apparently, tacrolimus had a higher safety relative to azathioprine in the study. Death has also occurred in studies involving the use of other immunosuppressants for NMOSD^8, 20, 25^. The dead cases in our study (1 in tacrolimus group and 1 in azathioprine group) developed life-threatening agent-induced severe infection, just like those in other reports^8, 20, 25^. Cautious infection monitoring and early application of anti-infective therapy are needed in patients who are receiving immunosuppressants.

It is generally thought that NMOSD is a disease mediated by humoral dysimmunity^1, 26^. However, further studies demonstrate that AQP-4 antibody, the hallmark of NMO, belongs to the IgG1 subgroup of immunoglobulins and requires the help of T lymphocytes in its production^27^. Thus, the rationale of tacrolimus treatment for the disease is based on the fact that tacrolimus exerts its effect on T helper cells to suppress production of various cytokines^28^, in turn reducing production of antibodies by B lymphocytes, just like many other kinds of humoral autoimmune diseases such as myasthenia gravis (MG). Moreover, AQP-4 antibodies also need T lymphocytes to open the blood–brain barrier in order to gain access to the CNS parenchyma^29, 30^. Activated T cells in the CNS create an environment for the facilitation of complement-mediated cytotoxicity (CDC) and antibody-dependent cellular cytotoxicity (ADCC) against their astrocytic targets^31, 32^. In consideration of these crucial roles, T cells do indeed play an important part in the induction of NMO lesions. Our data provided the evidence that suppressing T cells by tacrolimus is another effective target in preventing relapses in patients with NMOSD, in addition to blocking B cells by rituximab.

However, our study is a retrospective study with a small size and a short follow-up period in a single medical center, and thus the biases are inevitable. A randomized, large scale control trial involving multiple centers and long-term follow-up is needed to further evaluate the efficacy and safety of tacrolimus in treating NMOSD.

## Methods

### Participants

We retrospectively reviewed a cohort of NMOSD patients who were receiving or had received oral tacrolimus from Tongji hospital of Tongji Medical College, Huazhong University of Science and Technology (HUST), via our electronic medical record systems from Jan 1, 2011 to Oct 1, 2016. All the patients included were diagnosed with NMOSD based on 2015 international consensus diagnostic criteria for neuromyelitis optica spectrum disorders^33^. The inclusion criteria involved patients who had been treated with tacrolimus for more than 6 months. Patients with previous exposure to other immunosuppressants or immunomodulators such as cyclophosphamide, azathioprine, mycophenolate mofetil, interferon-beta and intravenous immunoglobulins G (IVIG), were also included in our study. Concomitant prednisone was regarded as concurrent treatment. All the patients were tested for AQP4-IgG antibody in the serum by live cell-based fluorescence-activated cell sorting (FACS) assays before tacrolimus treatment. The titer of AQP4-IgG was analyzed using a combination of specimen dilution ratios and fluorescence intensities. NMOSD patients who received azathioprine for more than 6 months over the same period were also included and analyzed at the same time.

#### Ethics Statement

Our study was approved by the Institutional Review Board at Tongji hospital of Tongji Medical College, HUST, with a waiver of informed consent obtained from the study subjects because of the retrospective and observational nature of the study and the de-identified data we used. This study was conducted in accordance with the Declaration of Helsinki. All methods were performed in accordance with the relevant guidelines and regulations.

#### Definitions and follow-up

Treatment efficacy was assessed by analysis of annualized relapse rate (ARR), and disability progression was evaluated by the Expanded Disability Severity Scale (EDSS) before and after the latest tacrolimus treatment. A relapse was defined as new neurological symptoms and signs lasting >24 h with or without a responsible lesion on gadolinium enhancing magnetic resonance imaging (MRI). ARR was calculated as the total number of relapses per patient–year. For the purpose of this analysis, a high titer of AQP4-IgG was defined as the titer in the serum above or equal to 1:64, and a titer below 1:64 or no detectable AQP4-IgG was defined as the low titer. We also concurrently recorded the dosage of tacrolimus, adverse effects and concomitant therapy during our treatment. We verified the accuracy of our data and obtained additional follow-up information through telephone calls.

#### Statistics

Statistical analysis was performed using IBM SPSS Statistics (version 20.0 for Windows; SPSS Inc., Chicago, IL). Mann-Whitney U test was used for continuous variables, and the Fisher’s exact test was used for categorical ones. We compared the ARR and EDSS before and after the treatment by the Wilcoxon signed rank test. The possibility of being free of relapses after treatment was compared between different titers of AQP-4 antibodies using log-rank test, and the risk was estimated using the Kaplan-Meier method. Adjusted and unadjusted associations of patient characteristics with the outcome were assessed with Cox proportional hazards regression model. Statistical significance was set at P < 0.05.

## Electronic supplementary material

Detailed clinical information of patients who received tacrolimus
