# Supplementary material for: Efficacy and safety of tacrolimus treatment for neuromyelitis optica spectrum disorder
Source: Sci Rep. 2017 Apr 11;7:831. doi: 10.1038/s41598-017-00860-y (PMC5429791; doi:10.1038/s41598-017-00860-y)
Supplement: Supplementary file 1 — Detailed clinical information of patients who received tacrolimus [file 41598_2017_860_MOESM1_ESM.pdf]

# Efficacy and safety of tacrolimus treatment for neuromyelitis optica spectrum disorder

Bo Chen[1], Qian Wu[1], Gaotan Ke[2], Bitao Bu[1,*]

[1]Department of Neurology, Tongji Hospital of Tongji Medical College, Huazhong University of Science and Technology, Wuhan, Hubei, China

[2]Department of Radiology, Tongji Hospital of Tongji Medical College, Huazhong University of Science and Technology, Wuhan, Hubei, China

*Correspondence and requests for materials should be addressed to B. T. B. (bubitao@tjh.tjmu.edu.cn)

| Patients | Gender | Onset age | Attack points (patients were prescribed tacrolimus at time 0, relapse after tacrolimus was marked by red) | Total number of attacks | Number of attacks before tacrolimus treatment | Number of attacks after tacrolimus treatment | ARR before tacrolimus | ARR after tacrolimus | EDSS before tacrolimus | EDSS after tacrolimus | AQP4-ab titer in serum |
|---|---|---|---|---|---|---|---|---|---|---|---|
| 25 | F | 55 | -13,-2,0,34 | 3 | 3 | 0 | 2.77 | 0.00 | 5 | 3 | 1:32 |
| 24 | F | 30 | -3,6,32 | 3 | 1 | 2 | 4.00 | 0.75 | 9 | 3 | 1:320 |
| 23 | F | 19 | -42,-11,27 | 2 | 2 | 0 | 0.57 | 0.00 | 4 | 3 | 1:100 |
| 22 | F | 6 | -38,-15,-8,0,25 | 4 | 4 | 0 | 1.26 | 0.00 | 3 | 0 | 1:32 |
| 21 | F | 22 | -3,17 | 1 | 1 | 0 | 4.00 | 0.00 | 6 | 1 | 1:32 |
| 20 | F | 22 | -31,-23,-18,-15,-11,-7,-5,-3,-1,7,13,15 | 11 | 9 | 2 | 3.48 | 1.60 | 3 | 3 | 1:10 |
| 19 | F | 21 | -4,-1,14 | 2 | 2 | 0 | 6.00 | 0.00 | 4 | 0 | 1:32 |
| 18 | F | 50 | -17,-1,14 | 2 | 2 | 0 | 1.41 | 0.00 | 4 | 3 | 1:32 |
| 17 | F | 51 | -17,-14,-3,0,13 | 4 | 4 | 0 | 2.82 | 0.00 | 6 | 1 | 1:32 |
| 16 | M | 47 | -31,-24,-13,-1,13 | 4 | 4 | 0 | 1.55 | 0.00 | 4 | 2 | 1:10 |
| 15 | F | 16 | -28,-25,-16,0,12 | 4 | 4 | 0 | 1.71 | 0.00 | 6 | 4 | 0 |
| 14 | F | 48 | -19,-1,4,12 | 3 | 2 | 1 | 1.57 | 1.00 | 3 | 3 | 1:64 |
| 13 | F | 35 | -5,-1,11 | 3 | 2 | 1 | 4.80 | 1.09 | 4 | 3 | 0 |
| 12 | F | 40 | -3,10 | 1 | 1 | 0 | 4.00 | 0.00 | 3 | 3 | 1:64 |
| 11 | F | 41 | -24,-20,-16,-8,-1,10 | 5 | 5 | 0 | 2.50 | 0.00 | 3 | 1 | 1:100 |
| 10 | F | 23 | -44,-20,-12,-1,9 | 4 | 4 | 0 | 1.09 | 0.00 | 6.5 | 1.5 | 1:10 |
| 9 | F | 50 | -4,-2,9 | 2 | 2 | 0 | 6.00 | 0.00 | 2 | 1 | 1:100 |
| 8 | F | 40 | -3,8 | 1 | 1 | 0 | 4.00 | 0.00 | 7 | 1 | 1:100 |
| 7 | F | 36 | -24,-18,-8,0,3,8 | 5 | 4 | 1 | 2.00 | 1.50 | 3 | **3** | 1:100 |
| 6 | M | 31 | -17,-2,7 | 2 | 2 | 0 | 1.41 | 0.00 | 3 | 1 | 1:32 |
| 5 | F | 19 | 23,0,7 | 9 | 8 | 1 | 1.52 | 1.71 | 4.5 | 10 | 1:100 |
| 4 | F | 22 | -2,7 | 1 | 1 | 0 | 6.00 | 0.00 | 3 | 0 | 0 |
| 3 | F | 17 | -99,-15,-11,0,5,6 | 5 | 4 | 1 | 0.48 | 2.00 | 5 | 3 | 1:64 |
| 2 | F | 49 | -5,6 | 1 | 1 | 0 | 2.40 | 0.00 | 7.5 | 3 | 1:10 |
| 1 | F | **22** | -2,6 | 1 | 1 | 0 | 6.00 | 0.00 | 5 | 2 | 1:320 |

| Patients | Dose of prednisone (6 months later)/mg | Time points of last follow-up (black) or first relapse after tacrolimus (red) | Previous therapy | Dose of tacrolimus/mg | Follow-up | Abbreviations |
|---|---|---|---|---|---|---|
| 25 | 0 | 34 | | 2 | | **ARR:** annualized relapse rate |
| 24 | 0 | 6 | | 3 | | **EDSS:** Expanded Disability Severity |
| 23 | 7.5 | 27 | | 2.5 | | **CTX:** cyclophosphamide |
| 22 | 5 | 25 | | 1 | | **PE:** Plasmapheresis |
| 21 | 0 | 17 | CTX*5d | 3 | | **AZA:** azathioprine |
| 20 | 10 | **7** | PE*3 | 3 | herpes zoster | **MMF:** mycophenolate mofetil |
| 19 | 10 | 14 | | 3 | | **IF:** interferon-beta |
| 18 | 0 | 14 | | 2 | | **IVIG:** intravenous immunoglobulins G |
| 17 | 10 | 13 | AZA*1m, | 2 | | **d:** day |
| 16 | 0 | 13 | | 3 | | **m:** month |
| 15 | 0 | 12 | IF*10m | 2 | | |
| 14 | 15 | 4 | IVIG*5d | 2 | | |
| 13 | 5 | **11** | | 3 | | |
| 12 | 0 | 10 | | 2 | | |
| 11 | 0 | 10 | PE*3 | 3 | | |
| 10 | 10 | 9 | AZA*18m, 2 | 3 | rash | |
| 9 | 10 | 9 | | 3 | | |
| 8 | 10 | 8 | AZA*2m, | 2 | hand tremor | |
| 7 | 15 | 3 | | 3 | | |
| 6 | 0 | 7 | | 3 | | |
| 5 | 0 | 7 | MMF*26M, 2 | 3 | death | |
| 4 | 7.5 | 7 | | 3 | | |
| 3 | 20 | 5 | IVIG*5d | 3 | | |
| 2 | 10 | 6 | | 3 | | |
| 1 | 10 | 6 | | 3 | | |
